# Characterization and Abundance of Plasmid-Dependent *Alphatectivirus* Bacteriophages

**DOI:** 10.1007/s00248-024-02401-3

**Published:** 2024-06-27

**Authors:** Boris Parra, Veronika T. Lutz, Lone Brøndsted, Javiera L. Carmona, Alejandro Palomo, Joseph Nesme, Vuong Van Hung Le, Barth F. Smets, Arnaud Dechesne

**Affiliations:** 1 Department of Environmental Engineering and Resource Engineering, Technical University of Denmark, Kongens Lyngby, Denmark; 2 Laboratorio de Investigación de Agentes Antibacterianos (LIAA), Departamento de Microbiología, Facultad de Ciencias Biológicas, Universidad de Concepción, Concepción, Chile; 3 Instituto de Ciencias Naturales, Facultad de Medicina Veterinaria y Agronomía, Universidad de las Américas, Concepción, Chile; 4 Department of Veterinary and Animal Sciences, University of Copenhagen, Copenhagen, Denmark; 5 School of Environmental Science and Engineering, Southern University of Science and Technology, Shenzhen, China; 6 Section of Microbiology, Department of Biology, University of Copenhagen, Copenhagen, Denmark; 7 Department of Biotechnology and Biomedicine, Technical University of Denmark, Søltofs Plads, Building 221, Kgs. Lyngby, 2800 Denmark

**Keywords:** Bacteria, Bacteriophages, Virus, Plasmid, Horizontal gene transfer, Conjugation, Antimicrobial resistance

## Abstract

Antimicrobial resistance (AMR) is a major public health threat, exacerbated by the ability of bacteria to rapidly disseminate antimicrobial resistance genes (ARG). Since conjugative plasmids of the incompatibility group P (IncP) are ubiquitous mobile genetic elements that often carry ARG and are broad-host-range, they are important targets to prevent the dissemination of AMR. Plasmid-dependent phages infect plasmid-carrying bacteria by recognizing components of the conjugative secretion system as receptors. We sought to isolate plasmid-dependent phages from wastewater using an avirulent strain of *Salmonella enterica* carrying the conjugative IncP plasmid pKJK5. Irrespective of the site, we only obtained bacteriophages belonging to the genus *Alphatectivirus*. Eleven isolates were sequenced, their genomes analyzed, and their host range established using *S. enterica*, *Escherichia coli*, and *Pseudomonas putida* carrying diverse conjugative plasmids. We confirmed that *Alphatectivirus* are abundant in domestic and hospital wastewater using culture-dependent and culture-independent approaches. However, these results are not consistent with their low or undetectable occurrence in metagenomes. Therefore, overall, our results emphasize the importance of performing phage isolation to uncover diversity, especially considering the potential of plasmid-dependent phages to reduce the spread of ARG carried by conjugative plasmids, and to help combat the AMR crisis.

**Supplementary Information:**

The online version contains supplementary material available at 10.1007/s00248-024-02401-3.

## Introduction

Conjugation is one of the main mechanisms for horizontal gene transfer (HGT) between bacteria, by which a donor cell directly transfers genetic material to a recipient cell [1]. About one-fourth of the thousands of plasmids that have been described are conjugative and contain the genes for their self-transfer [2]. Some of these plasmid genes encode for a type 4 secretion system (T4SS), which is a membrane-spanning proteinaceous complex structure which traverses the bacterial envelope. Conjugation systems are major subfamilies of T4SS. They contain a channel through which protein–DNA complexes can be translocated [3]. Notably, some proteins of the T4SS can serve as a receptor for a group of viruses known as plasmid-dependent (bacterio)phages, making the bacterial host harboring a conjugative plasmid susceptible to this phage group [4].

Antimicrobial resistance (AMR) is a major public health threat [5]. Since conjugation is an important mechanism contributing to the rapid dissemination of antimicrobial resistance genes (ARG) [6], targeting this process is a new promising strategy to help combat AMR [7]. Conjugative plasmids can be classified according to their incompatibility group, which is based on replication initiation protein sequences. Plasmids of the incompatibility group P, N, and W are self-transmissible and broad-host-range plasmids that often carry multiple antibiotic resistance determinants [8]. They are ubiquitous mobile genetic elements often isolated from wastewater treatment plants, manure, and soils [9] and are able to transfer and replicate in virtually all Gram-negative bacteria [10]. These plasmids usually carry modules dedicated to plasmid addiction, which ensures killing of plasmid-free segregants, and central control regions, which minimizes their burden imposed upon the host cell [11]. Moreover, even in the absence of antibiotics, it has been demonstrated that resistance plasmids can persist among bacterial populations after hundreds of generations [12]. Therefore, they are very relevant targets to prevent dissemination of AMR.

One poorly documented aspect of plasmid ecology is the degree to which bacteria carrying conjugative plasmids are subject to predation by plasmid-dependent bacteriophages. A number of bacteriophages lytic against bacteria carrying IncP, IncN, or IncW plasmids have been isolated from sewage (i.e., PRR1, PRD1, PR3, L17, PR4, Lu221, and Hi226), without a systematic effort to describe abundance and diversity of these phages [13, 14]. Most of such phages belong to the genus *Alphatectivirus*, that are dsDNA, icosahedral lipid-containing phages [15]. They are also known as PRD1-like phages, as PRD1 is the prototypal member of the group. They are broad host range phages, because they infect diverse plasmid-bearing bacteria such as *S. enterica*, *E. coli*, *P. putida*, *Pseudomonas aeruginosa*, *Proteus mirabilis*, *Vibrio cholera*, and *Acinetobacter calcoaceticus* [16].

*Alphatectivirus* were mainly isolated in the 1970s, but recent works described novel isolates [17–19]. Our aim was to demonstrate that *Alphatectivirus* are the dominant IncP-dependent bacteriophages in wastewater and to assess their distribution across contrasting environments to identify their main reservoir. We hypothesized that their primary source is the gut of warm-blooded animals, as is the case for other plasmid-dependent phages, including several genogroups of F-specific RNA coliphages [20]. In this work, we report the isolation and characterization of eleven *Alphatectivirus* phages. We also demonstrate the high abundance of this group of plasmid-dependent bacteriophages in wastewater using both culture-dependent and culture-independent approaches and search their genomes in metagenomic datasets from several types of environments.

## Materials and Methods

### Bacterial Strains and Plasmids

We isolated plasmid-dependent phages using *Salmonella enterica* subsp. *enterica* serovar Typhimurium BAA-2828™ (Strain MHM112) carrying the conjugative IncP plasmid pKJK5. This strain is an avirulent derivative of ATCC® 14,028™ cured of all its plasmids [21]. This strain was used because the abundance of *Salmonella* phages in wastewater is low compared to that of other Enterobacteriaceae [14]. The conjugative plasmid pKJK5 is a 54,383 bp broad-host-range genetic element that confers resistance against tetracycline and trimethoprim [22]; therefore, cultures were always grown in LB broth with tetracycline (10 µg mL^−1^). For host range determination (i.e., infectiveness against diverse bacteria carrying conjugative plasmids from several Inc groups), we used *S. enterica*, *E. coli*, and *P. putida* carrying plasmids from IncF, IncH, IncN, IncP, IncW, or IncX groups as models (Table 1). The strains were generated by surface mating, as described in He et al. [23].

**Table 1:** Conjugative plasmids used in this study. Antibiotic resistance determinants carried by each plasmid are included. The plasmids are carried by *Salmonella enterica* MHM112, *Escherichia coli* K12, or *Pseudomonas putida* KT2440

Plasmid	Inc group	Antibiotic resistance	Accession number
R1drd19	IncF	Ampicillin, chloramphenicol, kanamycin, streptomycin, and sulfonamides	NA. Derepressed version of plasmid R1, whose sequence is available at NZ_KY749247
pOXA436	IncH	Temocillin, piperacillin, ceftazidime, aztreonam, ertapenem, and sulfonamides	KY863418.1
drR27	IncH	Nalidixic acid and tetracycline	NA. Derepressed version of plasmid R27, whose sequence is available at NC_002305.1
pKM101	IncN	Ampicillin and rifampicin	NA. Derivative of R46, whose sequence is available at AY046276.1
RP4	IncP-1 alpha	Tetracycline, ampicillin, and kanamycin	NA. Identical to RK2, whose sequence is available at BN000925.1
pKJK5	IncP-1 epsilon	Tetracycline and trimethoprim	AM261282.1
pB10	IncP-1 beta	Amoxicillin, streptomycin, sulfonamides, and tetracycline	AJ564903.1
R388	IncW	Trimethoprim and sulfonamide	NC_028464.1
R6K	IncX	Ampicillin and streptomycin	LT827129

### Isolation of Plasmid-Dependent Phages

Samples were obtained from the influent of municipal wastewater treatment plants (WWTP) in Southern Sweden: Ryaverket, Visby, and Sjolunda. The samples were collected twice within a single week of the Fall 2020 using 24 h flow-proportional sampling and were kept refrigerated at 4 °C until processing within 10 days of collection. More details can be found elsewhere [23]. The samples were centrifuged at 8000 × g for 45 min at 4 °C, and then, the supernatant was collected to remove big particles and most bacterial cells. To remove the remaining bacterial debris while retaining the viral fraction, the supernatant was passed through a sterile 25-mm Whatman glass fiber membrane with a pore size of 0.22 μm (Sigma-Aldrich). These samples were collected in sterile 50-mL tubes and stored at 4 °C.

Phage isolation was done by the classical double layer agar (DLA) method [24]. For this, overnight cultures of *S. enterica* (pKJK5) were prepared in LB broth supplemented with tetracycline and incubated at 25 °C and 120-rpm shaking. Then, an aliquot of 100 µL from the overnight culture was inoculated in LB broth supplemented with tetracycline and incubated (25 °C and 120 rpm) to achieve the mid-exponential phase. Aliquots of 100 µL of this bacterial suspension were mixed with 100 µL of filtered environmental samples and 3 mL melted (50 °C) soft-LB agar (0.5%) supplemented with CaCl_2_ (final concentration 5 mM). After overnight incubation of the plates at 25 °C, single plaques were taken from each sampling location and harvested in 500 µL SM buffer (100 mM NaCl, 8 mM MgSO_4_, 50 mM Tris-HCl, pH 7.5). Then, the isolates were purified three times by DLA and sequential isolation. Subsequently, we checked if the isolates were plasmid-dependent by DLA with the plasmid-free strain *S. enterica* MHM112.

### Phage Characterization

#### Sensitivity to RNase and Chloroform

The sensitivity to RNase of the isolated phages was determined by DLA, in which RNase was added to the bottom agar at a final concentration of 10 µg mL^−1^. The resistance to chloroform of the isolates was determined by adding it at final concentration of 10 or 100 µL mL^−1^ in 200 µL of phage suspensions. Then, mixtures were incubated 60 min at 25 °C and phage viability was determined by DLA. All assays were done by triplicate.

#### Host Range

Host range (i.e., infectiveness against diverse bacteria carrying plasmids from several Inc groups) was determined using *S. enterica*, *E. coli, *and *P. putida* carrying IncF, IncH, IncN, IncP IncW, or IncX plasmids (Table 1). We inoculated a mixture of an overnight culture and a phage suspension from a range of four decimal dilutions for 20 min before being dropped (20 µL each) on LB agar in triplicate. The appearance of plaques in the lawn after overnight incubation at 25 °C was indicative of the phage ability to cause lytic infection. When too many plaques to count appeared for the fourth decimal dilution, experiments with the next two dilutions were performed.

#### Sequencing and Genome Analysis

DNA from phage suspensions was extracted with the Purelink Viral RNA/DNA mini kit (Invitrogen) and sequenced at the University of Copenhagen on the paired-end 2 × 250 bp Illumina sequencer HiSeq 2500 platform. The raw sequences were primarily handled with CLC Genomics Workbench and then the read quality was checked using FastQC version 0.11.8. The read trimming and genome assembly was done with the online tool PATRIC v3.6.12 at default parameters [25]. Retrieval of publicly available genomes similar to our newly assembled phage genomes was done using BLASTn v.2.15.0 + with default parameters and considering only complete genomes. Also, the genomic sequences published by [26], who re-sequenced most of the described *Alphatectivirus* genomes, were downloaded and used as references. Pairwise comparisons of the nucleotide sequences were conducted using the Genome-BLAST Distance Phylogeny (GBDP) method with settings recommended for prokaryotic viruses in the online resource Virus Intergenomic Distance Calculator (VIRIDIC) [27]. In addition, we constructed a phylogenetic tree using Tree Building Online Resource (VICTOR) web service (https://victor.dsmz.de) [28].

The annotation of the genomes was done using Phage Commander for rapid identification of bacteriophage genes using multiple gene identification programs [29, 30]. Phage Commander runs a bacteriophage genome sequence through nine gene identification programs (and an additional program for identification of tRNAs), such as RAST [31] and GeneMarks. tRNA genes were searched using the ARAGORN webserver. To infer the functions of the predicted genes, BLASTx was used with the parameter options set to default values, and the *E* value threshold set to 10^−4^.

### Environmental Search of *Alphatectivirus*

#### *Alphatectivirus *Distribution in Metagenomes

To determine the habitat preference of *Alphatectivirus*, we screened 182 public metagenomic datasets from diverse geographical regions: the Netherlands, China, Denmark, USA, Sweden, Japan, Germany, Spain, Singapore, and Austria; and diverse environments, such as human gut (*n* = 25), bird feces (*n* = 40), influent (*n* = 17) and basin (*n* = 23) of municipal WWTPs, surface water (*n* = 29), ground water (*n* = 24), and drinking water treatment plants (DWTP) (*n* = 24). Accession numbers of metagenomes are given in the Supplementary File. For comparison, we also determined the abundance of *Bacteroides* phages crAssphage in the metagenomes, as they are by far the most abundant phages in the human gut microbiome [32]. Reads from all metagenomics datasets were trimmed and filtered, including adapters removal, using Trimmomatic v.0.38 (threshold quality of 30 (SLIDINGWINDOW:4:30) and minimum length of 50 (MINLEN:50)). Mapping was conducted using bbmap v38.96 with minimum identity of 90% (0.9).

For the mapping of *Alphatectivirus*, we used the sequences of 20 phages: PRD1, PR3, PR4, PR5, L17, PR772, BCE1, LNA9, CSP1, PKJ.Ry.20.1, PKJ.Ry.20.2, PKJ.Ry.20.3, PKJ.Sj.20.1, PKJ.Sj.20.2, PKJ.Sj.20.3, PKJ.Vi.20.1, PKJ.Vi.20.2, PKJ.Vi.20.3, PKJ.Vi.20.4, and PKJ.Vi.20.5. For the mapping of Tectiviridae, we used 12 more sequences of phages from other genera in the family: *Betatectivirus*: Bam35, GIL16, AP50, Wip1, Sole, Sato, GIL01, and GIL02; *Gammatectivirus*: GC1; *Deltatectivirus*: Forthebois and WheeHeim; *Epsilontectivirus*: Toil.

For the mapping of crAssphage, we used the prototypical crAssphage described by Dutilh et al. [33], deposited in GenBank under accession code NC_067194.1 and BK010471 (formerly NC_024711.1) and recently renamed as *Carjivirus communis* [34].

#### Detection of *Alphatectivirus *in Domestic and Hospital Wastewater by qPCR

To determine the abundance of *Alphatectivirus* in hospital and domestic wastewater, we obtained samples from the influent of two Danish WWTP, Hillerød (domestic) and Herlev (hospital). Five mL was centrifuged at 8000 × *g* for 15 min at 4 °C and the supernatant was filtered at 0.22 μm. The flow-through virome was concentrated using Amicon® ultrafiltration membranes (100 kDa, Millipore Sigma). Then, DNA was extracted using Purelink Viral RNA/DNA mini kit and quantified using NanoDrop (Thermo Fisher Scientific) and Qubit BR dsDNA (Thermo Fisher Scientific).

Primers were designed to detect *Alphatectivirus* using the Primer3 program with default parameters. We used the most conserved genes within *Alphatectivirus* phages according to Saren et al. (2005): five genes without any base substitution, gene 11 (encoding a phage DNA packaging ATPase, ACLAME 1116), gene 12 (encoding a hypothetical protein), gene 13 (encoding a DNA packaging protein), gene 17 (encoding a phage packing DNA injection protein, ACLAME 1522), and gene 18 (encoding a putative phage transcription terminator protein, ACLAME 1242). We obtained 3 primer pairs that were checked for specificity *in silico* using BLAST tool. We ensured that the selected primers do not amplify other organisms, specifically other phages in the Tectiviridae family. Also, we checked that the selected primers amplified all the phages in the *Alphatectivirus* genus described by Saren et al. (2005). These primer pairs were ordered and checked experimentally using the genome of phage PRD1 as template. This phage was kindly provided by Matti Jalasvuori from University of Jyväskylä, Finland. PCR was performed in a total volume of 25 µL per sample with 2.5 µL of PCR Buffer (10×), 0.5 µM of each primer, 12.5 mM each dNTP, 50 mM MgCl2, 5 U/µL Phusion High-Fidelity Polymerase, and 1 µL DNA template. The cycling conditions were 94 °C for 10 min, followed by 40 cycles of denaturation at 94 °C for 30 s, annealing at 58 °C for 1 min, and elongation at 72 °C for 1 min. Amplicons were checked by agarose electrophoresis on 1% gel. Based on this, we determined that the best primer pair was P13_2F (CCCCATTAATTGGCTTATCGTC) and 13_2R (TACTCCGAAGTGACGGCAT), because no primer dimers were observed, and this pair amplified the control most efficiently.

For the qPCR, the final volume of each reaction was 10 µL, including 5 µL of the FastStart Essential DNA Green Mastermix 2X (Roche), 0.5 µM of each primer and 1 µL of template DNA. This DNA was standardized by dilution of both samples to 2 ng/µL. The PCR program was set as follows: a cycle of pre-heating at 94 °C for 10 min, followed by 40 cycles of denaturation at 94 °C for 30 s, annealing at 58 °C for 1 min, and elongation 72 °C for 1 min. The reaction was done in duplicate, including a negative control with ultrapure water. It was performed in a LightCycler 96 Real-Time PCR System. A standard curve with tenfold dilutions of PRD1 DNA was done and Ct values were extrapolated to this curve after the qPCR.

#### Enumeration of IncP-Dependent Phages in Hospital and Domestic Wastewater by DLA

To compare the abundance determined by qPCR to the number of infective IncP-dependent virions, we performed triplicate DLA on the same domestic and hospital wastewater samples and *S. enterica* MHM112 (pKJK5) was used as the target strain. Prior to their use, as described above, the samples were centrifuged and filtered to remove bacteria and other big particles while keeping the virome. After DLA, plates were incubated at 25 °C overnight, and plaques were counted.

## Results and Discussion

### Phage Isolation and Characterization

Wastewater samples contained 10^2^–10^3^ plaque-forming units of IncP-dependent virions per milliliter (PFU/mL). From these samples, we isolated eleven IncP plasmid-dependent phages: PKJ.Ry.20.1, PKJ.Ry.20.2, and PKJ.Ry.20.3 from Ryaverket; PKJ.Sj.20.1, PKJ.Sj.20.2, and PKJ.Sj.20.3 from Sjölunda; and PKJ.Vi.20.1, PKJ.Vi.20.3, PKJ.Vi.20.4, and PKJ.Vi.20.5 from Visby (Table 2). They are DNA phages, as indicated by their resistance to RNase in DLA, and they are sensitive to chloroform in broth, which indicates that they may be lipid-containing phages. We extracted and visualized their genomes by electrophoresis, showing that they consisted of a single molecule of approximately 15 kb.

Although the number of sequenced phage genomes in databases is constantly rising, only a relatively small number are well-characterized and taxonomically classified [35]. Genomic sequences of our isolated phages were submitted to GenBank with accession numbers mentioned in Table 2. A search against the GenBank nucleotide database found that the phages belong to the *Alphatectivirus* genus in the Tectiviridae family in the Kalamavirales order [36]. This family also includes the genera *Betatectivirus*, *Gammatectivirus*, *Deltatectivirus*, and *Epsilontectivirus* [37–39]. Although these genera share similar virion morphology (viral particles with double-layered icosahedral capsids) and genome architecture (single molecule of linear double-stranded DNA of 15–18 kilobases in length and a two-segment arrangement, with the first segment of genes on the reverse strand and the second, larger one on the forward strand), they have no sequence similarity, lifestyle, or host range [40]. *Betatectivirus* are lysogenic phages infecting *Bacillus* strains, *Gammatectivirus* infect *Gluconobacter* strains, *Deltatectivirus* infect *Streptomyces* strains, and *Epsilontectivirus* infect *Rhodococcus* strains [36, 41]. Only *Alphatectivirus* are plasmid-dependent phages that infect bacteria carrying IncP, IncW, or IncN conjugative plasmids.

**Table 2:** Genomic characteristics of isolated phages. Related *Alphatectivirus* are included

Phage	Bacterial host for isolation	Plasmid (Inc) for isolation	Genome size	GC content (%)	Place of isolation	Accession number
PKJ.Ry.20.1	*S. enterica* MHM112	pKJK5 (IncP)	14,756	48.4	Sweden	PP495474.1
PKJ.Ry.20.2	*S. enterica* MHM112	pKJK5 (IncP)	14,655	48.4	Sweden	PP495475.1
PKJ.Ry.20.3	*S. enterica* MHM112	pKJK5 (IncP)	14,747	48.2	Sweden	PP495476.1
PKJ.Sj.20.1	*S. enterica* MHM112	pKJK5 (IncP)	14,831	48.1	Sweden	PP495477.1
PKJ.Sj.20.2	*S. enterica* MHM112	pKJK5 (IncP)	14,875	48.2	Sweden	PP495478.1
PKJ.Sj.20.3	*S. enterica* MHM112	pKJK5 (IncP)	14,822	48.1	Sweden	PP495479.1
PKJ.Vi.20.1	*S. enterica* MHM112	pKJK5 (IncP)	14,897	48.2	Sweden	PP495480.1
PKJ.Vi.20.2	*S. enterica* MHM112	pKJK5 (IncP)	14,830	48.3	Sweden	PP495481.1
PKJ.Vi.20.3	*S. enterica* MHM112	pKJK5 (IncP)	14,837	48.3	Sweden	PP495482.1
PKJ.Vi.20.4	*S. enterica* MHM112	pKJK5 (IncP)	14,935	48.6	Sweden	PP495483.1
PKJ.Vi.20.5	*S. enterica* MHM112	pKJK5 (IncP)	14,806	48.1	Sweden	PP495484.1
PRD1	*P. aeruginosa*	RP1 (IncP)	14,927	48.1	USA	M69077, NC_001421.2, AY848689
PR3	*P. aeruginosa*	RP1 (IncP)	14,937	48.2	Australia	AY848685
PR4	*P. aeruginosa*	RP1 (IncP)	14,954	48.3	Australia	NC_007451.1, AY848686
PR5	*P. aeruginosa*	RP4 (IncP)	14,939	48.2	Canada	AY848687
L17	*E. coli*	RP1 (IncP)	14,935	48.3	UK	AY848684
PR772	*Proteus mirabilis*	R772 (IncP)	14,946	48.3	South Africa	AY441783, AY848688
BCE1	*Burkholderia cenocepacia*	–	14,800	48.2	–	OK041468
LNA9	*P. putida*	RP4 (IncP)	14,342	48.3	Australia	OM630588
CSP1	*Burkholderia contaminans*	pBCO-1 (IncP), pBCO-2, and pBCO-3	14,942	48.4	Australia	OQ674210

**Fig. 1:**
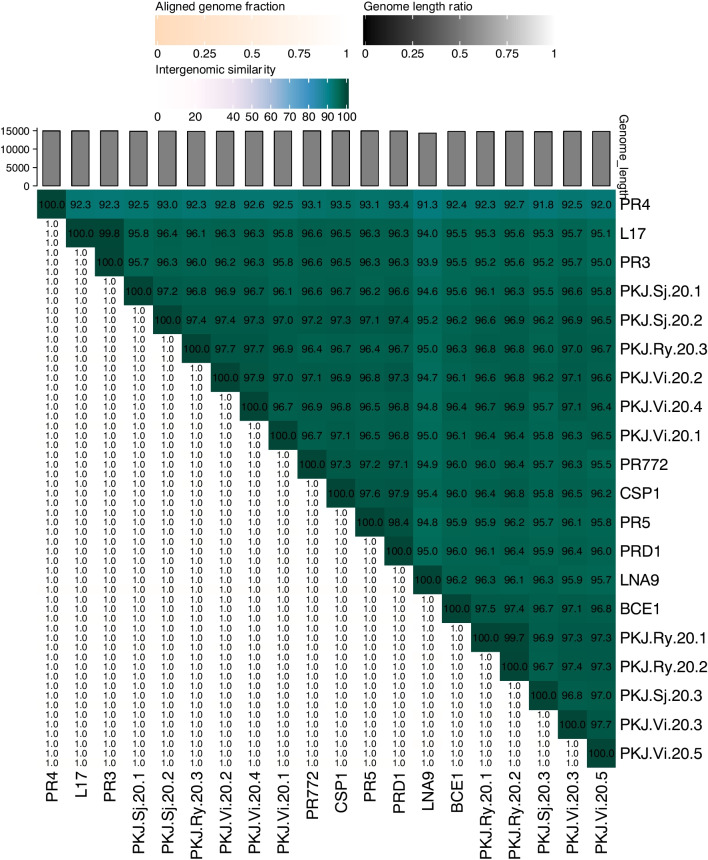
Differences in the nucleic acid sequences of the isolated plasmid-dependent phages and related *Alphatectivirus*. Pairwise genomic identity between genomes was determined with VIRIDIC. Aligned genome fraction and genome length ratio are 1.0 for all comparisons

**Fig. 2:**
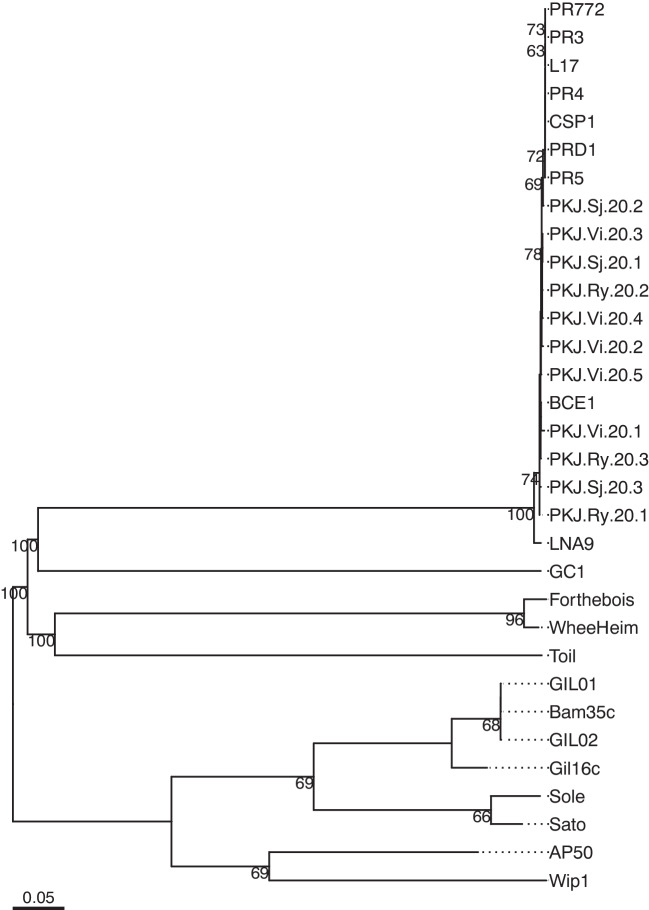
Whole genome-based phylogeny of our isolated phages and related phages in the family Tectiviridae. The phylogeny was inferred using VICTOR, based on the formula D0. The numbers above branches are the GBDP pseudo-bootstrap support values from 100 replications

The highest similarity in whole genomic sequences between our isolates and other *Alphatectivirus* is between PKJ.Ry.20.1 and BCE1 (97.5%). Although the latter has not officially been classified as an *Alphatectivirus*, it clearly belongs to this group according to its high genomic similarity to other members of this genus (Fig. 1) and by whole genome-based phylogeny (Fig. 2). It is unknown whether this phage is plasmid-dependent, but it is likely so since it was isolated using *Burkholderia cenocepacia*, and *Burkholderia* strains are known hosts of conjugative plasmids. Indeed, recently, Stanton et al. [18] described the *Alphatectivirus* phage CSP1, isolated using a strain of *Burkholderia* carrying three conjugative plasmids, one of them classified as IncP.

We found that our isolated phages present low diversity, with similarity to each other higher than 95.7% (Fig. 1). Generally, the genome sequences of the *Alphatectivirus* are highly similar, with overall identities between 89.3% and 99.8%. The lowest similarity is between PR4 and PKJ.Vi.20.5. Using the ANI 95% cut-off for species definition, the phage PKJ.Vi.20.5 is considered a novel *Alphatectivirus.* Therefore, this genus includes PR4-like phages, PRD1-like phages, and PKJ.Vi.20.5. Recently, Quinones-Olvera et al. [19] proposed the expansion of the genus from two to twelve species, after they isolated and described 51 *Alphatectivirus* from diverse environments. However, since these sequences were not deposited in the NCBI GenBank when preparing this manuscript, they are not included in our analysis.

Those with the highest identity are PR3 and L17 (99.8%), despite the large geographical distance between their isolation source (Australia and the UK, respectively). In our case, the phages PKJ.Ry.20.1 and PKJ.Ry.20.2 are likely clonal since their genomes are 99.7% similar (not shown), and they were isolated at the same location. However, the phage PKJ.Ry.20.2 has 62 nucleotides more than phage PKJ.Ry.20.1 at the beginning of the sequence. This may be because *Alphatectivirus* genomes have inverted terminal repeat sequences (ITR) and a replication-priming protein covalently linked to the 5′ termini [26], which might create a variation of genome lengths after sequencing due to the difficulty in determining the first 5′-end.

Remarkably, despite the differences in the time and place of isolation of *Alphatectivirus*, the genomes of these isolates are very similar. In this regard, Saren et al. [26] proposed a hypothesis that we share: *Alphatectivirus* have an optimal genome-level organization and structure, in which any change decreases fitness. These authors calculated free energies for the folded tectiviral ssDNAs, demonstrating that they were much lower than for randomly generated ssDNAs, reflecting a selective pressure that keeps tectivirus genomes unique at the nucleotide level as well as the protein level. The annotation of the genomes of our isolated phages predicted the presence of 31 putative proteins and no tRNA gene, like for others *Alphatectivirus* [26]. Twenty-eight (90.3%) putative proteins were detected on the negative strand and three (9.7%) on the positive strand. Twenty-six (83.9%) were annotated as functional proteins, while the others have unknown functions. Furthermore, no sequences related to integrase, virulence factors, antibiotic resistance, or any other harmful genes were identified.

### Host Range

**Table 3:** Host range of isolated *Alphatectivirus*. Bacterial hosts are listed with the plasmids they carried and the corresponding Inc group. We tested all 11 phage isolates, showing the same results. We showed the average of the infectivity of phages in PFU/mL

Inc group	Bacterial host	PFU/mL
F	*E. coli* K12 (R1drd19)	0
H	*S. enterica* MHM112 (drR27)	0
H	*E. coli* K12 (drR27)	0
H	*E. coli* K12 (pOXA436)	0
N	*S. enterica* MHM112 (pKM101)	1.4 × 10^9^ ± 4.5 × 10^0^
P	*P. putida* KT2440 (RP4)	1.5 × 10^9^ ± 4.7 × 10^0^
P	*P. putida* KT2440 (pKJK5)	1.2 × 10^9^ ± 3.3 × 10^0^
P	*S. enterica* MHM112 (pKJK5)	1.3 × 10^9^ ± 3.8 × 10^0^
P	*E. coli* K12 (pB10)	1.3 × 10^9^ ± 2.8 × 10^0^
P	*E. coli* K12 (RP4)	1.2 × 10^9^ ± 3.3 × 10^0^
P	*E. coli* K12 (pKJK5)	1.3 × 10^9^ ± 3.7 × 10^0^
W	*S. enterica* MHM112 (R388)	1.3 × 10^9^ ± 3.7 × 10^0^
X	*E. coli* K12 (R6K)	*0*

All isolated phages infected bacteria carrying plasmids belonging to the IncP, IncN, and IncW groups and could not form plaques using IncA/C, IncF, IncH, and IncX plasmid-carrying bacteria as hosts (Table 3). This plasmid specificity pattern is consistent with the described *Alphatectivirus* [42]. IncP, IncN, and IncW plasmid have in common that their pili are all straight and rigid and are constitutively synthetized, in comparison of other Inc groups encoding flexible pili that can be constitutively or repressed synthetized [8].

In terms of bacteria hosts, our isolated phages were able to form plaques on plasmid-bearing *P. putida*, *S. enterica*, and *E. coli*, which is also consistent with the bacterial host specificity of described *Alphatectivirus* in pseudomonads and *Enterobacteriaceae* [16]. This confirms that no secondary receptor on the surface of the bacteria affects the infection capacity of these phages; they only depend on proteins of the mating pair formation system.

### Abundance of *Alphatectivirus *in Metagenomic Datasets

In our complete dataset of 6.0 × 10^9^ metagenomic reads from different sources, we detected only 26 reads assigned to *Alphatectivirus*, while 272 reads were assigned to Tectiviridae and 7.1 × 10^5^ reads were assigned to crAssphage (Table 4). Even after correcting for the ca. 7-fold difference in genome size between *Alphatectivirus* and crAssphage (≈15 kbp versus ≈97 kbp, respectively), crAssphage remained largely more abundant. Yet, among the 182 metagenomes we screened, we detected *Alphatectivirus* in 6, Tectiviridae in 40, and *CrAssphage* in 112 (Table SI).

**Table 4:** Detection of Tectiviridae, *Alphatectivirus,* and crAssphage in metagenomic datasets

Sample	Reads	Reads assigned to Tectiviridae	%	Reads assigned to *Alphatectivirus*	%	Reads assigned to CrAssphage	%
Human gut	4.1 × 10^8^	0	0	0	0	4.2 × 10^5^	1.0 × 10^−1^
Bird feces	1.2 × 10^9^	96	7.7 × 10^−8^	2	1.6 × 10^−9^	1.9 × 10^4^	1.5 × 10^−3^
Influent WWTP	2.8 × 10^8^	20	7.1 × 10^−6^	20	7.1 × 10^−8^	2.4 × 10^5^	8.7 × 10^−2^
WWTP	9.0 × 10^8^	18	2.0 × 10^−6^	0	0	2.4 × 10^4^	2.7 × 10^−3^
Surface water	1.7 × 10^9^	108	6.4 × 10^−6^	4	2.4 × 10^−9^	5.9 × 10^3^	3.5 × 10^−4^
Ground water	1.1 × 10^9^	24	2.3 × 10^−6^	0	0	10	9.4 × 10^−7^
Drinking water	4.6 × 10^8^	6	1.3 × 10^−6^	0	0	0	0
Total	6.0 × 10^9^	272	4.5 × 10^−6^	26	4.3 × 10^−9^	7.1 × 10^5^	1.2 × 10^−2^

The distribution of *Alphatectivirus* across environments contrasts with that of crAssphage (Table 4). Indeed, the latter displays often high (albeit very variable across individuals) abundance in human gut metagenomes and a decreasing abundance trend across influent WWTP, reactors of WWTP, bird feces, and freshwater metagenomes. Such distribution is consistent with the human gut as preferential habitat and a progressive decline after excretion, as expected for crAssphage [43]. On the other hand, *Alphatectivirus* were not detected in human gut samples while they were occasionally detected in other habitats. Therefore, we conclude that the main source of *Alphatectivirus* is not the human gut. This absence of strong association with the gut might be explained by the low occurrence of plasmids from the IncP, IncW, or IncN groups in the human gut microbiome [44]. We also determined that unlike some F-dependent coliphages, avian intestinal microbiomes are not the preferential habitat of *Alphatectivirus*. We conclude that this analysis failed to identify a preferential habitat for these plasmid-dependent phages.

We also note that searching phages in metagenomes is not without limitations. Indeed, most of these datasets were prepared, prior to sequencing, to study prokaryotic communities rather than viruses (i.e., metagenome samples generally are concentrated by centrifugation and the supernatant is discarded; as a result, a large proportion of phage virions are omitted). Therefore, these datasets would mostly include viral sequences from phage-infected bacterial cells (i.e., virocells). Consequently, phage detection will depend on the timing of the phage life cycle and how long the phage remains associated with its bacterial host. Because *Alphatectivirus* are strictly lytic, their abundance likely appears lower than those lysogenic phages. For instance, it has been demonstrated that phages are poorly represented in metagenomic datasets from wastewater [45] and human gut [46].

Lack of *Alphatectivirus* detection in metagenomic data has also been reported for virome samples. In a recent study, Quinones-Olvera et al. [19] searched for *Alphatectivirus* in a viromic data from a wastewater sample, where the concentration of such phages had been measured at 10^3^ PFU/mL by culture-dependent methods. No reads were detected, even though, the authors followed a protocol generally suitable for virome sequencing (i.e., filtration and concentration of the viral fraction by 100-fold, before performing DNA extraction and bulk sequencing). Moreover, they searched for *Alphatectivirus* in many metagenomic datasets representing different sequencing depths, locations, and sample processing methods. Consistent with our results, they demonstrated that over 75% of the samples contained 5 or fewer reads assigned to *Alphatectivirus*. They hypothesized that a combination of a low relative abundance, small genome size, and highly polymorphic population might be responsible for these results.

### Abundance of *Alphatectivirus *in Wastewater by qPCR

The enumeration of phages by qPCR indicates a concentration of *Alphatectivirus* of 3.9 × 10^4^ copies/mL and 8.1 × 10^4^ copies/mL, in domestic and hospital wastewater, respectively (Table 5). The abundance determined by DLA in the same samples indicates a concentration of IncP-dependent phages of 1.7 × 10^2^ PFU/mL and 3.1 × 10^3^ PFU/mL, respectively. This result is of the same order of magnitude as that estimated by Quinones-Olvera et al. [19] by DLA in fresh influent from two wastewater sites in Massachusetts, USA.

**Table 5:** Enumeration of *Alphatectivirus* by qPCR and IncP-dependent phages by DLA

Sample	qPCR (copies/mL)	DLA (PFU/mL)
Domestic WWTP	3.9 × 10^4^ ± 4.5 × 10^1^	1.7 × 10^2^ ± 2.1 × 10^1^
Hospital WWTP	8.1 × 10^4^ ± 1.7 × 10^1^	3.1 × 10^3^ ± 4.0 × 10^2^

Our results by qPCR enumerations are more than one log larger than the DLA ones. This difference could be because qPCR assay is more sensitive than DLA. Quantitative PCR, unlike DLA, enumerates gene copies irrespective of the context in which these exist and thus even if they are not associated with an infective virion. Therefore, the DNA present in defective viral particles or in virocells will be counted by qPCR but not DLA. Therefore, after the sample preparation (i.e., filtration to remove bacteria and concentration of viral particles), we postulate that qPCR is a robust approach for the detection and quantification of *Alphatectivirus.*

Most importantly, our results by qPCR and culture-dependent methods confirmed that *Alphatectivirus* plasmid-dependent phages are indeed very abundant in wastewater environments. Therefore, this makes relevant the need to carry out more studies on *Alphatectivirus* and other plasmid-dependent phages, which are natural predators of bacteria carrying conjugative plasmids, especially in environments such as wastewater, largely recognized as hotspots for HGT [47–49].

## Conclusion

We isolated plasmid-dependent *Alphatectivirus* from wastewater, a habitat where we demonstrated that they are abundant, using both culture-dependent and molecular approaches. However, they are under-detected in metagenomes, where they always appear at very low or undetectable levels. This complicates the determination of their main reservoir and emphasizes the importance of isolating phages to uncover diversity.

### Supplementary Information

Below is the link to the electronic supplementary material.

Supplementary Material 1

Supplementary Material 2

## Data Availability

No datasets were generated or analysed during the current study.
