# Supplementary material for: Characterization and Abundance of Plasmid-Dependent Alphatectivirus Bacteriophages
Source: Microb Ecol. 2024 Jun 27;87(1):85. doi: 10.1007/s00248-024-02401-3 (PMC11211187; doi:10.1007/s00248-024-02401-3)
Supplement: Supplementary file 1 — Supplementary Material 1 [file 248_2024_2401_MOESM1_ESM.pdf]

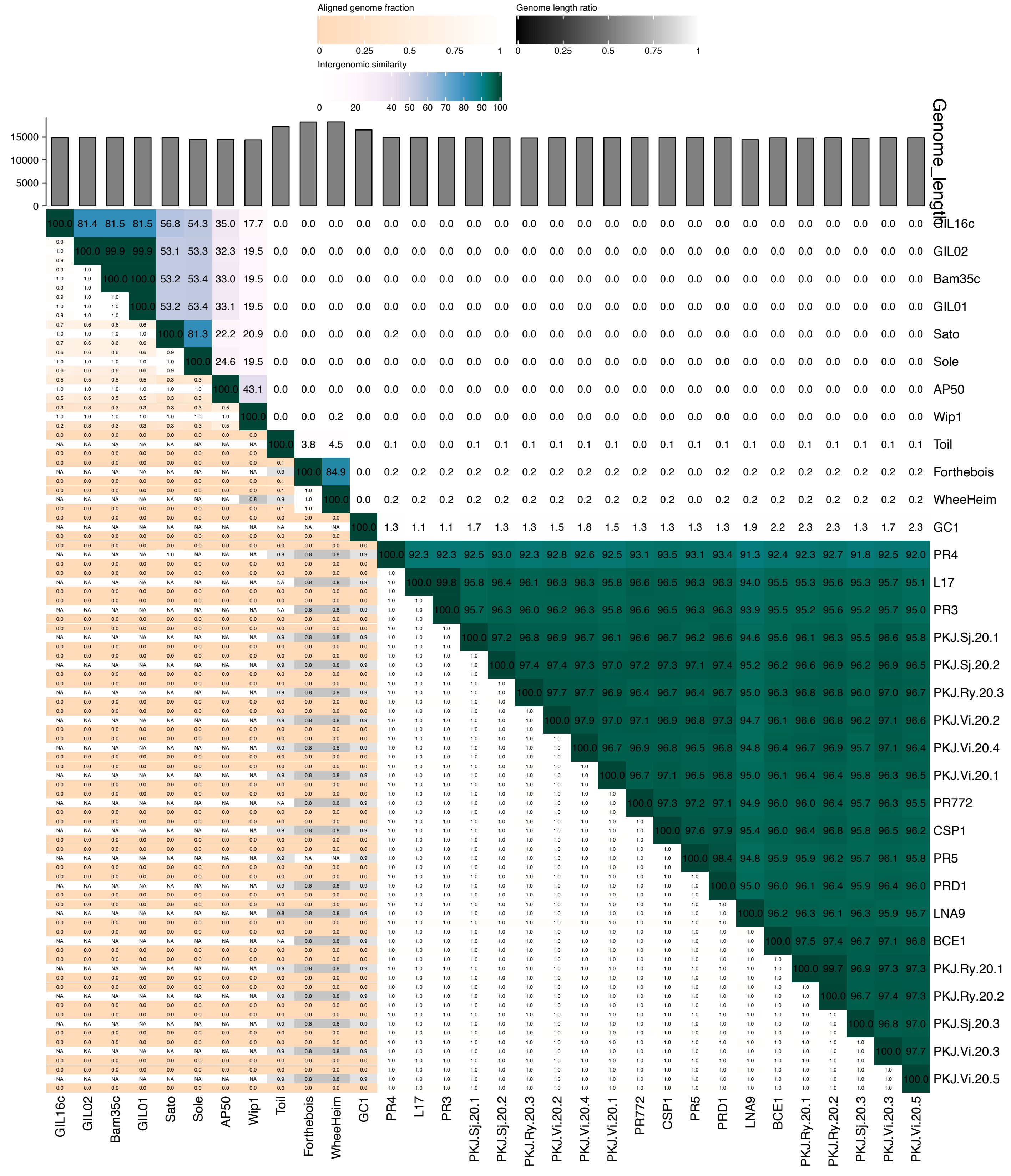

Figure S1. Differences in the nucleic acid sequences of the isolated plasmid-dependent phages and related Tectivirus. Sequence identity between pairwise genomes was determined with VIRIDIC.
